# Minimum Platoon Set to Implement Vehicle Platoons in the Internet of Vehicles Environment

**DOI:** 10.3390/s25227066

**Published:** 2025-11-19

**Authors:** Haijian Li, Xing Liu, Zonglin Han

**Affiliations:** College of Metropolitan Transportation, Beijing University of Technology, Beijing 100124, China; xingliu@emails.bjut.edu.cn (X.L.); herbert911@163.com (Z.H.)

**Keywords:** vehicle platooning, minimum platoon set, internet of vehicles environment, dynamic evolution process

## Abstract

Vehicle platoons offer significant benefits in connected vehicle environments, including reduced travel time, increased throughput, mitigated congestion, and lower energy consumption. To adapt to dynamic traffic conditions, platoon formations must be adjusted flexibly; this process is facilitated by traffic management centers via real-time control and cloud-based data transmission. Given communication constraints, we propose a minimum information set that is sufficient to maintain and adjust platoon formations and that supports data storage, computation, and representation of state changes within platoons. This set comprises two components: the Property Set and the Instruction Set. The Property Set collects vehicle-level and platoon-level attributes, whereas the Instruction Set, which includes communication and control subsets, enables formation maintenance and adjustment. We design a series of algorithms structured along timeline-based and task-based frameworks to specify transition rules and task execution modes across states, thereby describing the complete life cycle of a platoon from independent driving through formation and reorganization to dissolution. Finally, we develop an integrated scenario algorithm and apply it to two representative cases: highway platooning and intersection merging and separation. The results indicate that the proposed Minimum Platoon Set has substantial potential for platoon management, providing a solid theoretical foundation and practical guidance for optimizing platoon control.

## 1. Introduction

Due to advances in the Internet of Vehicles (IoV) and automation technologies, vehicles can now travel in platoons with minimal inter-vehicle distances. Platooning is a promising approach for mitigating traffic congestion, reducing energy consumption, and enhancing road safety. Truck platooning is likely to be among the earliest applications of road vehicle automation [1].

Extensive research on vehicle platooning has been conducted, with researchers obtaining empirical data from road tests in several cases. Prior studies have considered various vehicle- and platoon-level attributes and functions in platooning applications. To maximize the benefits of platooning, platoons should be formed by automated vehicles regardless of their manufacturers. Meanwhile, real-world roadways will likely carry mixed flows of human-driven and autonomous vehicles in the coming decades. Therefore, we have also contemplated the potential composition of platoons comprising both of these categories. The sole prerequisite for this composition is vehicle connectivity, i.e., vehicles being capable of networked communication within a vehicular network environment. This composition is not restricted by vehicle type or control mechanisms. To effectively manage the vehicles thus composed and enable them to perform platoon functions, it is imperative, under networked conditions, to clearly define and specify which information is used for intercommunication. This ensures coordination and stability within the platoon. Furthermore, considering potential challenges posed by latency and packet loss, to prevent excessive data transmission and information redundancy, it is necessary to select and determine specific information for bundled transmission. We refer to this non-redundant, essential information required for platoon functionality as the Minimum Required Information. In order to present this information effectively, serving the needs of platoon systems, platoon management, and platoon research, we have established the Minimum Platoon Set. It should be noted that the composition of the Minimum Platoon Set not only encompasses physical quantities but also includes elements such as platoon command instructions and platoon identification information. Hence, using the proposed set, a demonstration of the whole life cycle dynamic evolution process of vehicle platoons in the Internet of Vehicles environment can be carried out.

The new generation of communication technology supports the development of the Internet of Vehicles (IoV) technology [2,3]. The IoV technology is increasingly becoming a tool for researchers to solve modern transportation problems [4,5].

A platoon refers to a group of vehicles traveling in the same lane at uniform speed with minimal spacing. The present research focuses on truck platoons, which have been applied in practical commercial applications [6]. Beyond commercial benefits, platooning offers significant transportation and environmental advantages. Platooning can improve road capacity, alleviate congestion and save energy [7].

The combination of the IoV and the platoon strategy is a promising research trend. The development of the IoV technology enables a group of vehicles with the same or similar destinations to achieve self-organization of vehicle flow, thus forming a flexible platoon to optimize individual driving behavior and system efficiency [8]. Heavy-duty vehicle platooning is more economical [9]. Additionally, vehicles within a platoon maintain nearly identical speeds, thereby reducing speed variability and improving overall traffic safety [10]. The impact of the platoon organization on the road capacity under the autonomous driving road system [11], platoon formation strategy [12], the communication connectivity of the platoon [1,13], and platoon operation control strategy [14,15,16] have been studied. Luo and Timmerman proposed a platoon control algorithm and gave a communication protocol [17,18].

The different application scenarios of vehicle platoons in the IoV environment have been studied by researchers. Refs. [19,20] focused on the intersection scenario. Ref. [21] studied the changing process in a single lane, and ref. [22] studied the scenario of multiple lanes. Refs. [16,23] studied platoon optimization and on-ramp control strategies.

Limited research has been conducted on platoon information sets. Ref. [24] studied numerical safe sets but did not mention a collection of other attributes, such as speed or platoon destination. Maiti et al. [25] merely mentioned several indicators.

In summary, extensive studies on vehicle platooning have been conducted considering different scenarios, including vehicle-infrastructure cooperative architecture and control strategy, conceptual description of the platoon and platoon organization, optimization, and platoon set. However, limited research exists on data types and logical algorithms for effectively describing platoon structures and behavioral rules. Moreover, no uniform standard has been established for the data types required by platoon leaders to achieve cooperative driving.

To address these gaps, this study focuses on platoon information management rather than control management or communication management, aiming to provide a standardized information framework for future platoon management systems. Unlike existing research that primarily emphasizes control algorithms or communication protocols, this paper approaches the problem from a system-level management perspective. Specifically, we identify and formalize the minimum essential information set required to achieve complete platoon functionality, serving as a foundational specification for the development and implementation of upper-level platoon management systems.

The main contributions of this paper are summarized as follows:

(1) We create the Minimum Platoon Set by integrating all the essential platoon-related attributes or data and functions. This Minimum Platoon Set not only fulfills the minimum requirements but also satisfies all essential elements.
(2) We demonstrate the application, universality, and effectiveness of the Minimum Platoon Set through algorithmic implementations and scenario-based validations.

The paper is organized as follows. Section 2 defines the Minimum Platoon Set for vehicle platoons. Section 3 introduces the complete life cycle dynamic evolution process of vehicle platoons using the Minimum Platoon Set. Section 4 presents the proposed Minimum Platoon Set in two typical scenarios and provides simulation-based experimental validation. Finally, Section 5 presents the conclusions and future directions.

## 2. The Minimum Platoon Set

By analyzing the existing research on vehicle platooning, the minimum set is proposed to describe the basic elements and fundamental attributes of a vehicle platoon. The implication of “minimum” is the minimum amount of information required to achieve the full platoon function, such as platoon merging, platooning and platoon splitting.

**Figure 1:**
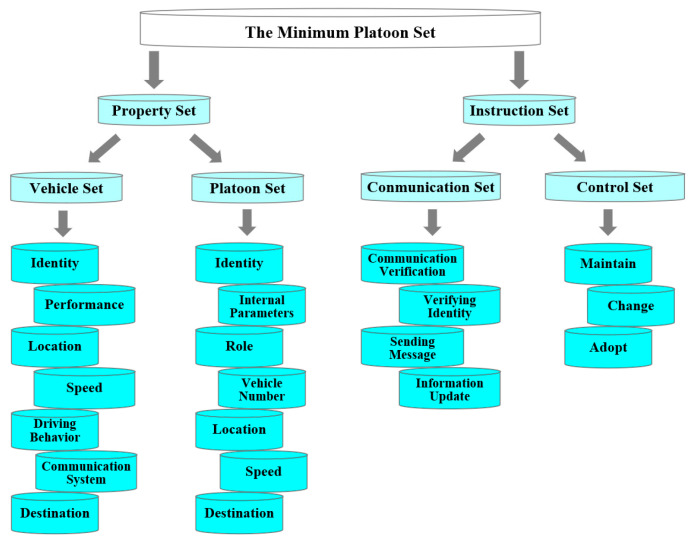
The minimum vehicle platoon set.

The entire Minimum Platoon Set is shown in Figure 1. The Minimum Platoon Set consists of measurable data, vehicle/platoon-relevant attributes, and operating instructions. It should be noted that only necessary attributes of the platoon or vehicles within the platoon are included in the Minimum Platoon Set. It comprises two parts: the Property Set and the Instruction Set. The Property Set includes the Vehicle Property Set (*VPS*) and the Platoon Property Set (*PPS*). The Vehicle Platoon Instruction Set (*VPIS*) comprises the Communication Set and the Control Set.

### 2.1. Vehicle Platoon Property Set

The Minimum Vehicle Platoon Set, denoted as *Y*, serves as the foundational framework for implementing platoon organization strategies. *Y* is defined as the union of a Property Set (*P*) and an Instruction Set (*I*):

(1) Y=(P,I)

Here, P={VPS,PPS} includes the Vehicle Property Set (*VPS*) and Platoon Property Set (*PPS*), while *I* = {communication, control} comprises the communication and control subsets. This integration enables the description of both static attributes (e.g., vehicle performance, platoon configuration) and dynamic operations (e.g., merging, splitting) within a unified structure.

The Vehicle Property Set (*VPS*) is presented as follows. For each vehicle i(i=1,2,3,…,n) in the system:

(2) $$VPS_{i}\;=[VID_{i},VP_{i},VL_{i},VS_{i},MDB_{i},VCS_{i},VD_{i}]$$

where,

(3) VP=[Maximum Acceleration,Maximum Deceleration,Maximum Speed,Maximum Grade]

(4) VL=[Longitude,Latitude]

(5) VD=[Destination]

Vehicles must be equipped with these seven standardized parameters to enable cross-manufacturer communication and support efficient platoon formation in the Internet of Vehicles (IoV) environment.

(1) ***VID*** (Vehicle ID): The vehicle ID is the unique identifier for connected vehicles accessing the internet. Each vehicle is assigned only one ID, emphasizing the uniqueness of its identity.
(2) ***VP*** (Vehicle Performance): The vehicle performance refers to the performance parameters of a vehicle, including maximum acceleration (*MA*), maximum deceleration (*MD*), maximum speed (*MS*), and maximum allowed climbing grade (*MG*). The detailed specification of *VP* = [*MA, MD, MS, MG*].
(3) ***VL*** (Vehicle Location): The vehicle location denotes the vehicle’s position on the road, specified by longitude and latitude coordinates. This information is one of the factors influencing the vehicle’s role in the platoon. For the specific vehicle role determination algorithm, see Section 3.
(4) ***VS*** (Vehicle Speed): The vehicle speed represents the real-time velocity of a vehicle, which is used for speed synchronization and dynamic adjustments within the vehicle platoon.
(5) ***MDB*** (Microscopic Driving Behavior): Microscopic driving behavior encompasses the detailed decisions and maneuvers performed by an individual vehicle during road operation, specifically including acceleration, deceleration, lane changing, and following behaviors.
(6) ***VCS*** (Vehicle Communication System): The vehicle communication system represents a vehicle’s capability to communicate with other vehicles and determines whether the vehicle can join or create a platoon. Notably, transmission delay time is a critical performance metric.
(7) ***VD*** (Vehicle Destination): The vehicle destination encompasses not only the ultimate destination but also all intermediate routes the vehicle is expected to traverse. Vehicles sharing common route segments can form a platoon. *VD* influences the choice of control strategy; see Section 3.

The above provides a comprehensive description of the vehicle set. Among these, *VID* and *VP* are classified as basic information. While basic information does not determine whether vehicles can form a platoon, it is utilized after a platoon has been established. In addition to the basic information components, *VL*, *VS*, *MDB*, *VCS*, and *VD* are categorized as judgmental information. These elements determine whether vehicles meet the requirements for forming a platoon and define their roles within the platoon.

The Platoon Property Set (*PPS*) is presented as follows. For each platoon *j* (*j* = 1, 2, …, m) in the system:

(6) $$PPS_{j}=[PID_{j},PIP_{j},PR_{j},PVN_{j},PL_{j},PS_{j},PD_{j}]$$

Among them,

(7) PIP=[Headway]

(8) PR=[Platoon leader,Platoon follower]

(9) PVN=[Platoon Maximum Number,Real-time Vehicle Number]

(10) PL=[Longitude,Latitude]

(11) PD=[Destination]

A platoon is an organizational form characterized by the cooperative operation of vehicles. It is a dynamic entity that undergoes continuous changes from formation to disbandment (see Section 3). While some attributes are updated in real-time, others remain relatively stable.

To achieve efficient and stable platoon operations, relatively stable information is classified as static attributes, while attributes requiring real-time updating are considered dynamic attributes. Based on this classification, the Minimum Platoon Set is divided into two categories.

First, the static dataset comprises relatively stable information such as Platoon ID (*PID*) and Platoon Internal Parameters (*PIP*), which are updated only when the platoon structure changes. This static data serves two purposes: providing clear and stable identification and establishing essential control parameter benchmarks for the platoon. Consequently, unnecessary frequent information exchanges are minimized, reducing communication overhead and enhancing operational stability. Importantly, the definition and management of static parameters depend on establishing stable and reliable foundational information. Only when this foundation is firmly established can smooth collaboration and information exchange among vehicles be ensured, thereby guaranteeing platoon safety and efficiency.

(1) ***PID*** (Platoon ID): The platoon ID is a unique identifier with a temporary lifespan. It can be assigned in two ways: either by the platoon control center or generated by the platoon leader during platoon formation. Once created, the platoon leader broadcasts it to all platoon followers.
(2) ***PIP*** (Platoon Internal Parameters): The platoon internal parameters include the platoon parameter setting (e.g., time headway or space headway). These parameters are subject to multiple constraints depending on operational conditions.
(3) ***PR*** (Platoon Role): The platoon role specifies the function a vehicle performs within the platoon. Each vehicle assumes only one role in the platoon, either as the platoon leader or a platoon follower.
(4) ***PVN*** (Platoon Vehicle Number): The platoon vehicle number includes two components: the maximum allowable number of vehicles in a platoon (*PMN*) and the real-time vehicle number in the platoon (*RVN*). Since the vehicle count is not updated in seconds or milliseconds, it is considered static data. The format of *PVN* is represented as *PVN* = [*PMN, RVN*].

Secondly, the dynamic dataset of the platoon is established. The dynamic dataset includes Platoon Location (*PL*), Platoon Speed (*PS*), and Platoon Destination (*PD*). Parameters such as *PL* and *PS* require real-time updates (on the order of seconds or milliseconds) to maintain accurate positional and speed information, thus ensuring operational safety and coordination within the platoon. The *PD*, however, is unique among dynamic attributes: although categorized as dynamic, it does not require millisecond-level real-time updates. Instead, *PD* is updated in an event-driven manner, occurring only when the platoon reaches the current adjacent destination node to confirm the subsequent adjacent destination. This integrated design of static and dynamic datasets effectively supports platoon operations under diverse scenarios, significantly improving the efficiency and accuracy of platoon information exchanges.

(5) ***PL*** (Platoon Location): The platoon location represents the driving position of the platoon, specified by the latitude and longitude coordinates of the platoon leader on the road map. The platoon has two types of location coordinates: absolute coordinates relative to the map (where it can be treated as a point particle) and relative positions within the platoon, with the platoon leader as the reference point. Therefore, each vehicle maintains relative horizontal and longitudinal position data.
(6) ***PS*** (Platoon Speed): The platoon speed is defined as a set that encompasses all types of velocities within the platoon or individual vehicles. It consists of two components: first, the platoon’s real-time speed (*PRS*), acceleration (*PAS*), and speed limit (*PSL*); and second, the speed (*VS*) and acceleration (*VAS*) of each individual vehicle. The platoon speed limit (*PSL*) is defined as the minimum speed limit (*VSL*) among all vehicles in the platoon, ensuring uniform operation. The platoon’s speed characteristics are represented as: *PS* = [*PAS, PSL, PRS*], where max-*PRS* = min{max (*VS*)}, max-*PAS* = min{max (*VAS*)}, and *PSL* = min(*VSL*).
(7) ***PD*** (Platoon Destination): The current adjacent destination node of the platoon is represented by *PD* (Platoon Destination). When the platoon arrives at this node, the next adjacent destination will be updated accordingly. Therefore, *PD* is classified as a dynamic attribute; however, it does not require real-time updating (e.g., at the millisecond level). Instead, it is event-driven, being updated only when the platoon reaches its designated destination node.

Applying the Minimum Platoon Set to heterogeneous vehicles enables platoon formation and coordination as an intelligent entity.

### 2.2. Vehicle Platoon Instruction Set

If the vehicle platoon possesses only internal attributes, it cannot fulfill the communication and control requirements between vehicles or platoons. Therefore, it is necessary to establish the Vehicle Platoon Instruction Set (*VPIS*), which defines communication and control criteria between vehicles or platoons. This instruction set, functioning as a vehicle platoon control language, works together with the Property Set to achieve complete platoon functionality.

The Vehicle Platoon Instruction Set (*VPIS*) has seven basic modules; *VPIS* is as follows:

(12) VPIS=[CV,VI,SM,IU,Maintain,Change,Adopt]

(1) ***CV*** (Communication Verification): The communication verification serves as a fundamental mechanism that enables vehicles or platoons to execute operational instructions. When the vehicles or platoons are “in communication,” they are able to perform coordinated actions for operational transformation. The core functionality of communication verification can be updated at millisecond intervals, ensuring rapid responsiveness in dynamic environments.
(2) ***VI*** (Verifying Identity): For vehicles, two aspects need to be confirmed. The first is whether the vehicle is the executor when instructions are being transmitted. The second is to determine its role within the platoon (whether it is a platoon leader or a platoon follower). For the platoon, instruction *VI* is used to select the executor.
(3) ***SM*** (Sending the Message): Use the *SM* instruction to facilitate information transmission. The transmitted information comprises basic property data and instruction data. For instance, the platoon leader transmits a message to the fourth platoon follower, containing the property data “platoon velocity is 60” and the instruction data “maintain.”
(4) ***IU*** (Information Update): Within a platoon, information is managed at two levels. The platoon leader maintains the comprehensive data for the entire platoon, while each platoon member retains its own local data. The platoon’s cluster heads update the overall platoon status by incorporating new information shared by other platoon leaders during merge and split operations. Additionally, platoon followers can locally update relevant dynamic data, based either on their direct perceptions or on instructions from the platoon leader (e.g., when cluster head assignments change).

The above four instructions (*CV, VI, SM, IU*) enable communication between vehicles and platoons, while the following three instructions (*Maintain, Change, Adopt*) control vehicle and platoon behavior.

There are two basic instructions for vehicles and one special instruction for the platoon: “*maintain*,” “*change*,” and “*adopt*.”

(5) ***Maintain:*** When vehicles execute this instruction, their state remains unchanged.
(6) ***Change:*** When vehicles execute this instruction, at least one of their states (position, condition, or both) changes.
(7) ***Adopt:*** When the platoon selects a formation (or changes platoon parameters) or when the platoon’s status changes, the platoon executes this instruction.

These seven basic instruction units form a comprehensive control instruction set, and all complex instructions can be derived from combinations of these basic units.

### 2.3. Using Format of the Vehicle Platoon Set

Exclusively using either the vehicle platoon property set or instruction set does not fully realize the functions or transformations of a platoon. Therefore, combining the utilization of both sets is necessary to achieve this goal.

The typical structure is as follows: [instruction, property].

Example 1: [*SM, PL*] refers to the platoon sending a message about the platoon position to the vehicles.

Example 2: [*Change*, *PVN*] refers to changing the platoon vehicle amount that will be used when a vehicle joins the platoon or leaves the platoon.

Example 3: [*IU*, *VPS*] refers to the Vehicle Property Set for updating information; when the platoon state changes, all properties of both the vehicle and the platoon need to be updated synchronously.

In this section, we introduce the combination of the vehicle platoon property set and instruction set. The integration of these properties and instructions enables the complex transformations of vehicles and platoons, as discussed in Section 3.

### 2.4. Supplementary Information

The internal authority of the vehicle platoon, information transmission direction, and assignment of information processing tasks need to be clarified.

The interpretation of the platoon instruction set varies according to the different roles within the platoon (platoon leader or platoon follower). The details are shown in Table 1.

For communication verification (*CV*) and information update (*IU*), the roles of platoon leaders and followers are the same. For verifying identity (*VI*), this instruction is used to select the platoon leader. Subsequently, platoon followers must verify their role and confirm the identity of the platoon leader. For the control instructions, the platoon leader functions as both the decision-maker and executor in adopting strategies, whereas the platoon followers serve solely as executors of the instructions.

Considering information security, information storage, computation of transmission, and energy consumption, the information of the Vehicle Property Set (*VPS*) is not shared with all participants in the platoon. Complete information needs to be shared with the platoon leader, while only information such as *VID*, *VL*, and *VCS* should be shared with all members of the platoon. The details are shown in Table 2.

Additionally, the update cycle of shared information must be clarified. Once set, the *PID* and *PIP* cannot be easily changed. The *PR* and *PVN* will be updated when there is a change in the platoon’s status, such as when the leader changes or a vehicle leaves the platoon. The platoon follows a predefined destination, and upon reaching it, the platoon’s status changes as the *PD* changes. The *PL* and *PS* need to be updated in real-time. The details are shown in Table 3.

Finally, it should be pointed out that the information transmission direction, information storage address, and data processing task assignment need to be further clarified, but this is not the focus of this paper.

Regarding instruction reliability, the framework provides inherent support for fault-tolerant operations through the following mechanism: When an instruction (*Change* or *Adopt*) is transmitted, the sender can monitor whether corresponding property changes (*PR*, *PVN*, *PL*, etc., as defined in Table 3) occur within an expected timeframe. If the expected update is not detected, *CV* can verify the communication link status. If *CV* confirms connectivity, the instruction can be retransmitted; otherwise, *IU* can be invoked to perform full state resynchronization. As shown in Table 1, both leaders and followers possess *CV* and *IU* capabilities, enabling bidirectional fault detection and recovery without requiring additional information elements beyond the proposed minimum set.

The final part of this section compares the proposed set with other investigator-related platoon studies to demonstrate the proposed set’s broad applicability to existing research and the possibility of future research as a fundamental theory.

Table 4 lists related studies on vehicle platoons. By comparing the main elements and functions required in each study with those in the proposed set, we verify the practical feasibility and universal applicability of the Minimum Platoon Set. This set serves as a fundamental research tool that ensures all necessary aspects are considered for platooning operations. While the current formulation is comprehensive, the framework can be extended to incorporate additional elements as new operational requirements emerge.

In Table 4, CACC (Cooperative Adaptive Cruise Control) represents an advanced cruise control system based on vehicle-to-vehicle (V2V) communication and sensor fusion. Its core function is to dynamically adjust the vehicle’s acceleration and following distance by real-time sharing of the lead vehicle’s motion state information (such as acceleration, speed, and position) in conjunction with the vehicle’s own sensor data (such as radar and cameras), thereby achieving high-precision, low-latency vehicle cooperative control. OBU (On-Board Unit) refers to an embedded electronic device integrated into the vehicle, serving as a core component of the vehicle-to-everything (V2X) network. It is responsible for enabling bidirectional communication and data processing between the vehicle and external entities (other vehicles, roadside units, and cloud platforms).

Table 5 demonstrates the feasibility of the instruction set by mapping core platooning functionalities to the proposed instructions. The communication set and control set correspond to the communication and control functions of the platoon. Communication architectures and platoon control models from related studies can be derived by combining the instruction set components.

### 2.5. Justification of Minimality and Sufficiency

The claim of “minimality” in the Minimum Platoon Set requires rigorous justification. This section demonstrates that the proposed set satisfies both necessity (each element is essential) and sufficiency (the set enables all essential platoon functions).

The justification follows a reverse-engineering approach: rather than arbitrarily selecting information elements, we systematically derive the required information set from the essential operational requirements of platoon management. The essential platoon operations, which are universally recognized in the field and comprehensively covered by Section 3.3, include: (1) platoon formation through vehicle or platoon merging, (2) platoon state changes such as lane changing and leader switching, and (3) platoon dissolution and separation.

Each element in the *VPS* and *PPS* is mapped to specific platoon functions that cannot be executed without it. Table 6 presents this mapping relationship.

Taken together, Table 6 demonstrates necessity. Each information element is required by at least one essential platoon operation, and removing any single element makes that operation infeasible. Sufficiency follows from the construction. The algorithms in Section 3.3 realize formation, state changes, and splitting using only the Property Set and the Instruction Set. No additional information is used. Therefore, the Minimum Platoon Set is both irreducible and complete for platoon management. No proper subset remains sufficient, and any superset is redundant for the essential functions considered.

Section 4.1 and Section 4.2 provide scenario demonstrations that walk through the platoon life cycle using the Minimum Platoon Set. These examples improve interpretability and do not change the set itself. Section 4.3 complements the logical argument with SUMO-based simulations that evaluate effectiveness and efficiency when the Minimum Platoon Set is instantiated in a simulation environment.

In the next section, we detail the life-cycle algorithms and show how they are applied in representative scenarios.

## 3. Platoon Complete Life Cycle Dynamic Evolution Scenario Demonstration

### 3.1. Timeline: The Complete Life Cycle Scenarios

The description of each scenario in the full life cycle of a platoon is based on the following assumptions:

(1) Vehicles are equipped with Cooperative Adaptive Cruise Control (CACC) functions and can communicate with each other through Vehicular Ad hoc Networks (VANETs) or Roadside Systems (RSSs) with vehicle-to-infrastructure communications.
(2) No dedicated lane exists for vehicle platooning on the road.
(3) All platoons are spontaneously formed on the road, and vehicles in the platoons are not required to have a common destination, ensuring greater applicability.

**Figure 2:**
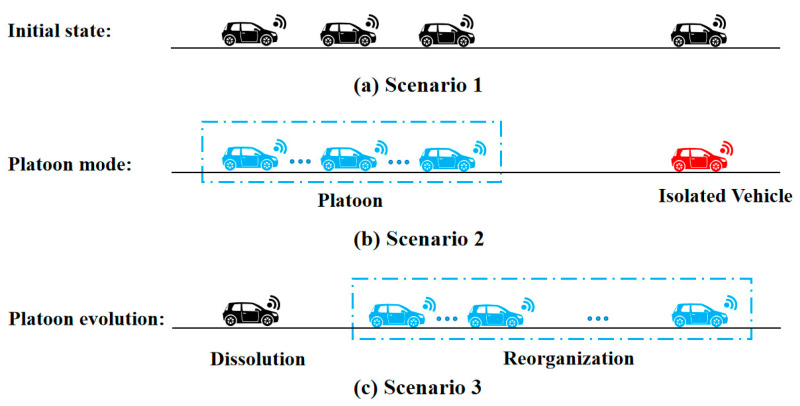
The complete life cycle of the vehicle platoon.

The complete life cycle evolution process of the vehicle platoon is illustrated in Figure 2.

In Scenario 1 (Initial state), all vehicles were driving autonomously, using onboard sensors or through the internet to receive information to form a platoon.

The change from Scenario 1 (Initial state) to Scenario 2 (Platoon mode) is due to the achievement of certain conditions, such as the distance between vehicles being less than a certain range, reaching the communication standard, and therefore enforcing some different tasks. In this section, only vehicles and platoons are discussed when they are already in a relatively stable scenario. In Scenario 2 (Platoon mode), the vehicles have completed the formation of a platoon, and all the vehicles follow the agreed control protocols, execute the instructions assigned by the platoon leader, and feedback on their information.

**Figure 3:**
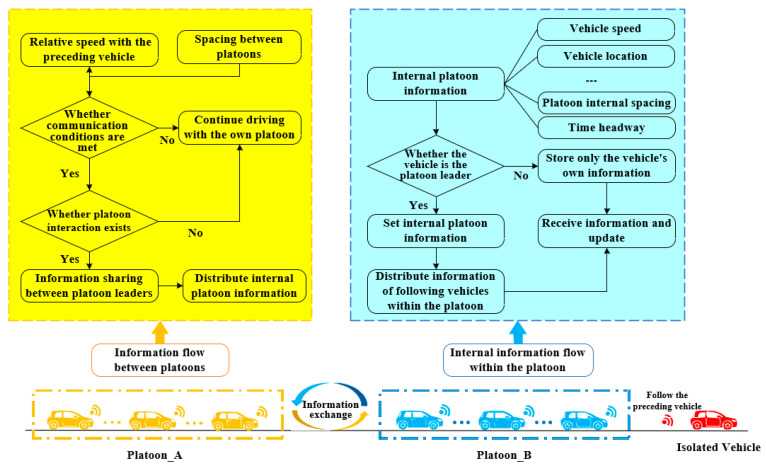
Information system workflow.

In Scenario 2 (Platoon mode), the specific forms of information flow between vehicles within the platoon, as well as the information flow between the platoon and other platoons or intelligent vehicles in the overall traffic flow environment, are shown in Figure 3. The information transmission logic is designed to enable efficient data flow while avoiding redundancy.

In Scenario 3 (Platoon evolution), the condition of the platoon will be changed. Vehicles may be reorganized to form a new platoon, or vehicles may leave the platoon to become isolated vehicles.

### 3.2. Taskline: Use Tasks to Achieve Scenario Transformation

The realization of the above scenario transformation requires the execution of different tasks at different time points by vehicles or platoons.

**Figure 4:**
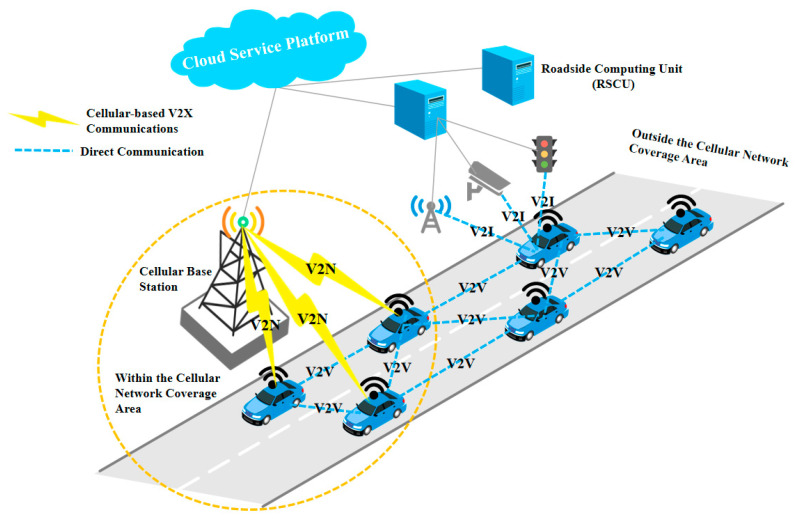
Communication of vehicles and roadside facilities.

**Figure 5:**
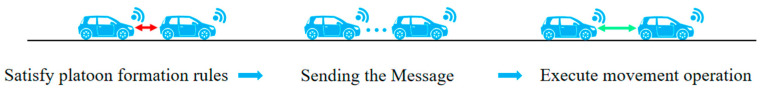
A descriptive task can be broken down into specific tasks to be performed.

All the tasks can be divided into two parts: one is information transmission tasks, and the other is platoon execution tasks. The information transmission tasks are shown in Figure 4. The platoon execution tasks are divided into scenario-changing tasks and specific tasks (involved Property Set update), as shown in Figure 5.

Specifically, as shown in Figure 2, the instruction used in the transition from Scenario 1 to Scenario 2 is “*Merge*”. In Scenario 2, some of the instructions that might be used include “change leader”. The transition from Scenario 2 to Scenario 3 requires the instruction “*Split*”. These scenario transition instructions are composed of specific instructions within the Vehicle Platoon Instruction Set. For example, the “*Merge*” instruction involves “*SM* (Sending the Message)” and “*Change*”. Furthermore, vehicles/platoons must also follow certain rules when executing the instructions in the Instruction Set.

The focus of the task line is to describe the dynamics of the platoon transformation, that is, the process of the transition between the discrete state and the platooning state. The vehicles and the platoon complete these transitions by executing certain instructions.

### 3.3. Algorithm: Protocol Criteria in Platoon Transformation

Vehicles and platoons must follow specific regulations when executing transformation instructions. The algorithms proposed in this section describe these regulations and demonstrate how platoon properties change after instruction execution, as well as the updates to the Vehicle Property Set (*VPS*) and Platoon Property Set (*PPS*).

A total of 5 algorithms are designed to describe the rules that vehicles or platoons need to follow when performing tasks, the instructions that need to be used in the evolution process, and the update of the set after executing the instructions. These 5 algorithms are divided into two categories. The first category focuses on describing the update of the set (marked as “update set”), and the second category contains specific instruction details (marked as “complete process”).

The platoon algorithm is divided into three categories according to the state of the platoon: the platoon merge algorithm, the platoon state transfer algorithm, and the platoon split algorithm.

**Figure 6:**
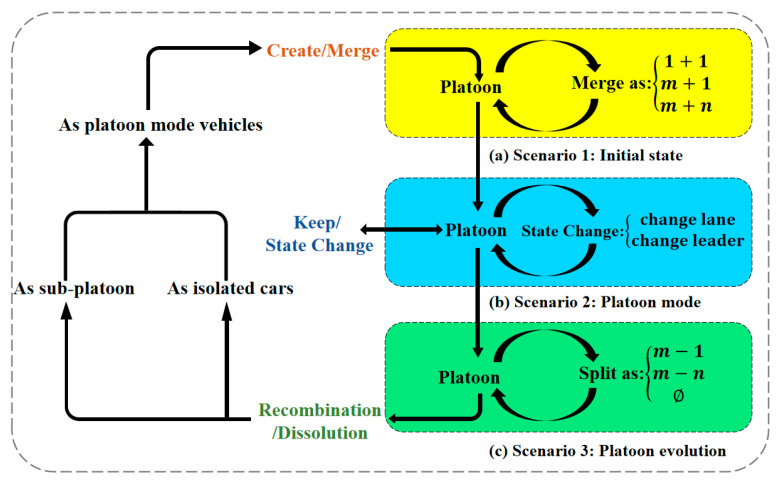
The function of the algorithms in changing the states of vehicles and platoons.

The relationship between the three algorithms is shown in Figure 6.

(13) $$Merge=\left\{\begin{array}{c} 1+1,\\ m+1,\\ m+n, \end{array}\right.\begin{array}{c} if\;vehicles\;merge\\ \;if\;vehicle\;and\;platoon\;merge\\ if\;platoons\;merge \end{array}$$

(14) $$State\;Change=\left\{\begin{array}{c} Change\;lane,\\ Change\;leader, \end{array}\begin{array}{c} update\;vehicle\;position\\ update\;platoon\;role \end{array}\right.$$

(15) $$Split=\left\{\begin{array}{c} m-1,\\ m-n,\\ Ø, \end{array}\begin{array}{c} if\;followers\;leave\\ \;if\;split\;into\;two\;new\;platoons\\ if\;platoon\;dissolution\;occurs \end{array}\right.$$

In the above explanation, *m* represents the number of vehicles currently in a platoon, while *n* indicates the number of vehicles in other platoons involved in the merging or splitting process. 1 represents a single independent vehicle. For instance, when merging with the current platoon of size *m*, the number of platoons increases by one unit, resulting in *m* + 1, with the number of vehicles in the platoon increasing. The Ø signifies the dissolution of a platoon, where all vehicles no longer remain within the same group, and after dissolution, no platoon objects are available for merging.

In the beginning, the vehicle acts as an isolated vehicle. After the “*Merge*” instruction in the initial state, the vehicle is converted to the platoon driving mode. In platoon mode, the “*State Change*” instruction is used to change the platoon state. The platoon executes a “*Split*” instruction, which results in platoon reorganization and platoon dissolution. The specific changes and the effect of the algorithms are shown in Figure 6.

**Figure 7:**
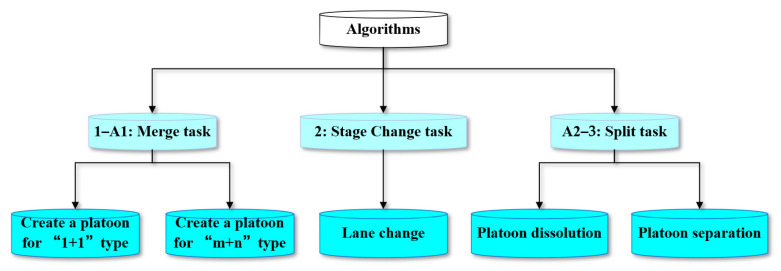
Schematic diagram of the algorithm logic structure.

Specifically, these ten algorithms are classified as follows. Algorithms 1 and A1 are specific algorithms for the “*Merge*” task. Algorithm 2 is designed for platoons in “*State Change*.” Algorithms 3 and A2 apply to the “*Split*” phase. The hierarchy and classification of the overall algorithms are shown in Figure 7.

**Figure 8:**
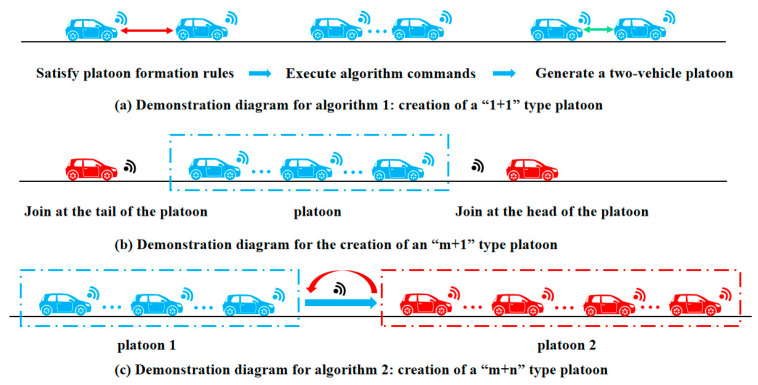
Algorithms 1 and 2 demonstration diagram.

Algorithm 1 is a fundamental platoon formation algorithm in which one connected vehicle and another connected vehicle form a two-vehicle platoon under specific rules, as illustrated in Figure 8a. In this algorithm, the property sets of the two vehicles are first defined and their communication capabilities are verified. If the communication requirements are met, the platoon leader and follower are selected based on the key judgment parameters within the vehicle property sets. The states of both vehicles are then updated through information transmission, and the consolidated updated information forms the Platoon Property Set (*PPS*), thereby completing the platoon formation.

Algorithm A1 is a platoon merging algorithm, with the detailed algorithm provided in Appendix A and illustrated in Figure 8c. Specifically, Algorithm A1 describes the merging process for constructing an “*m + n*” type platoon. First, the property sets of the platoons to be merged (Platoon_A and Platoon_B) are defined, and it is verified whether the merged platoon (Platoon_C) can accommodate the total number of vehicles from both platoons; if the capacity is exceeded, an empty platoon is returned. Otherwise, the *VI* function is employed to select the leader and follower vehicles for the merged platoon, followed by information transmission (*SM*) to synchronize the platoon attributes (e.g., *PID, PIP, PL, PS*). Finally, information update (*IU*) is executed to integrate the data, thereby forming a new Platoon Property Set and completing the merging process.

Additionally, the merging process for constructing an “*m* + 1” type platoon, as illustrated in Figure 8b, which represents the merging of vehicles and platoons, is structurally analogous to the algorithmic steps of Algorithms 1 and A1, and is therefore not elaborated further.
**Algorithm 1:** Create a platoon for the “1+1” type
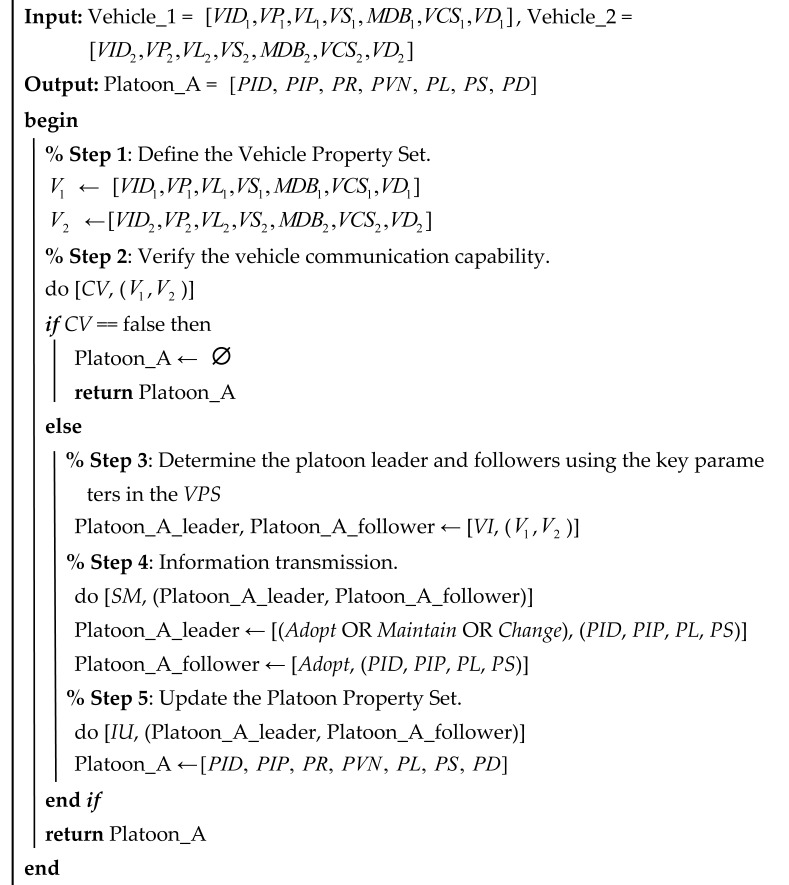


The platoon state change algorithm encompasses platoon parameter updates, changes in vehicle roles within the platoon, and lane-changing operations. Algorithm 2 details the lane change procedure for a platoon. Initially, the lane change request for Platoon_A is processed via information transmission (*SM*); if lane change is not permitted, the platoon retains its current state. Otherwise, the platoon leader issues a “*Change*” command while the follower vehicles execute the “*Adopt*” command. Subsequently, an information update (*IU*) operation adjusts the Platoon Location (*PL*) to reflect the new state after the lane change, and the updated platoon (new_Platoon_A) is returned.
**Algorithm 2:** Lane change
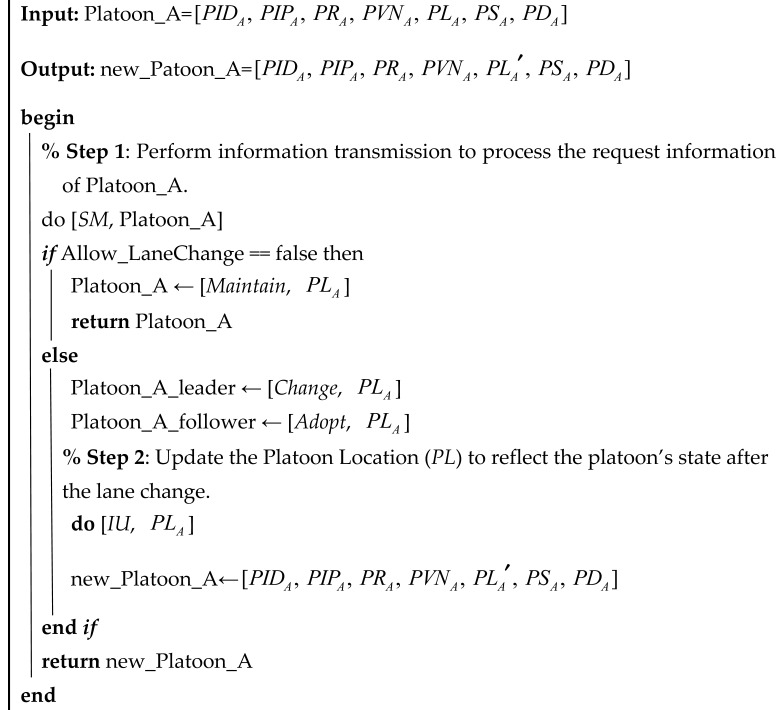


Algorithm A2 delineates the platoon dissolution process. Initially, information transmission (*SM*) is performed to handle the dissolution request for Platoon_A. If the split is not permitted (i.e., Allow_Split is false), Platoon_A maintains its current state. Otherwise, the algorithm sequentially extracts each vehicle from Platoon_A (Vehicle_1 through Vehicle_i), considering each as an isolated vehicle, and returns these individual vehicles, thereby dissolving the platoon. For the detailed content of Algorithm A2, please refer to Appendix B.

Algorithm 3 outlines the procedure for platoon separation. Initially, the separation request from Platoon_A is processed via information transmission (*SM*). If the request is denied (i.e., *Allow_Split* is false), the platoon remains unchanged. Otherwise, the platoon’s identification and vehicle number parameters (e.g., *PID, PVN*) are updated to remove Vehicle_***i***, which is treated as an isolated vehicle. Finally, the algorithm returns both the updated platoon (new_Platoon_A) and the separated vehicle (Vehicle*_**i***).
**Algorithm 3:** Platoon separation
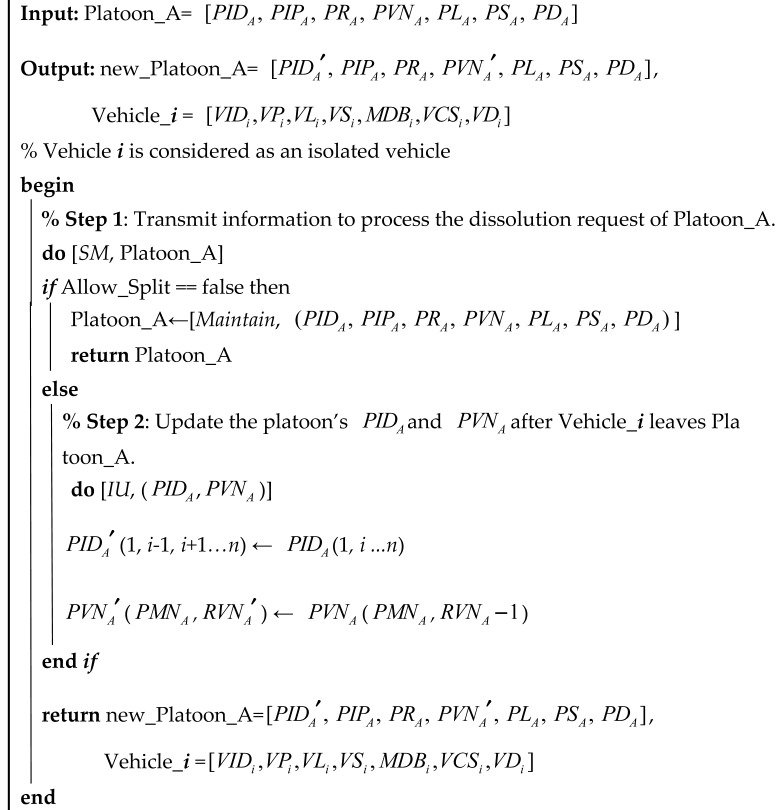


The algorithms presented in this section are intended to illustrate examples of essential information updates, as well as the conditions and rules governing their application. They can be used to illustrate the dynamic evolution of the platoon lifecycle, support effective platoon management and communication, and serve as templates for developing both fundamental and innovative scenarios for platoon evolution.

## 4. The Application of Minimum Platoon Set in Two Scenarios

This section demonstrates the self-organizing process of vehicles and platoons using the Minimum Platoon Set and the logical algorithms describing the entire platoon life cycle operation. Two typical scenarios are selected: a basic section of a four-lane highway/expressway (speed limit of 120 km/h) and a four-entrance intersection (without lane widening). In these scenarios, the complete platoon life cycle evolution is implemented by invoking the different algorithms described in Section 3.3.

### 4.1. Scenario 1: Basic Freeway Section

**Figure 9:**
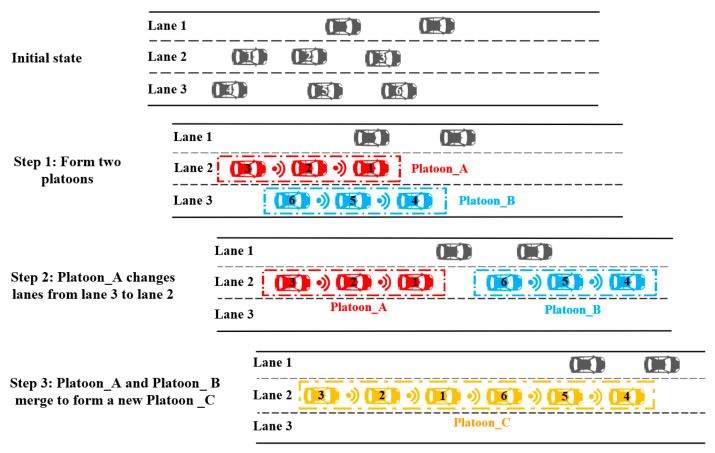
The dynamic evolution process in the basic section.

In the basic freeway section, the scenario assumes that eight vehicles with communication functions are equipped with the Internet of Vehicles technology. They are in a discrete state at the beginning. At a certain point during operation, eight vehicles comprised three platoons, initiating platoon formations. Subsequently, at a particular moment, Platoon_A and Platoon_ B were required to merge into a new platoon. Platoon_A continued to travel in the second lane, while Platoon_B transitioned from lane 3 to lane 2. Upon the completion of the platoon transformation, Platoon_A and Platoon_B coalesced into a new Platoon_C. A dynamic demonstration of the whole process is shown in Figure 9.

The changes in the vehicle set and platooning the whole life cycle are as follows:

**In the initialization phase (as isolated vehicles):**

(1) The six vehicles on lanes 2 and 3 have distinct and unique vehicle codes, which are simplified here as 1, 2, 3, 4, 5, and 6.
(2) Assume that all vehicles are equipped with the same Vehicle Communication System (*VCS*), exhibit similar Microscopic Driving Behavior (*MDB*), and have the same Vehicle Performance (*VP*).
(3) All vehicles have predetermined destinations to ensure that vehicles will travel a certain distance as a platoon, namely, $VD_{1},\;VD_{2},\;VD_{3},\;VD_{4},\;VD_{5}$, and $VD_{6}$.
(4) The speeds of vehicles 1 to 6 are 88, 76, 111, 90, 102, and 100 km/h, respectively. The positions of the vehicles are denoted as $(Vx_{1},Vy_{1}),(Vx_{2},Vy_{2})\;,(Vx_{3},Vy_{3}),(Vx_{4},Vy_{4})\;,(Vx_{5},$$Vy_{5})\;$ and $\left(Vx_{6},Vy_{6}\right)$.

**In the platooning phase** **:**

(5) The platoon set has been created, and the platoon obtains its unique ID (*PID*) from the roadside system, simplifying Platoon_A and Platoon_B.
(6) The Platoon Internal Parameter (*PIP*) is set by the platoon leader; for example, the headway is 0.5 s, and so on.
(7) Platoon Role (*PR*) includes the platoon leader and platoon follower. Assume that Vehicle_1 plays the role of platoon leader of Platoon_A, Vehicle_4 plays the role of platoon leader of Platoon_B, and the rest of the vehicles act as platoon followers. Therefore, Platoon_A’s *PR* is (1; 2, 3), while Platoon_B’s *PR* is (4; 5, 6), indicating the roles of the platoon leader and followers within each platoon.
(8) The Platoon Vehicle Number (*PVN*) is composed of the Platoon Maximum Vehicle Number (*PMN*) and the Real-time Vehicle Number (*RVN*). For instance, Platoon_A has a *PMN* of 8 and an *RVN* of 3, resulting in a *PVN* of (8, 3). Similarly, Platoon_B has a *PMN* of 8 and an *RVN* of 3, with its *PVN* also being (8, 3).
(9) The platoon position is determined by the platoon leader. Assume that Platoon_A has the position of $\left(Px_{A},Py_{A}\right)$, and Platoon_B has the position of $\left(Px_{B},Py_{B}\right)$.
(10) The Platoon Speed (*PS*) is composed of the Platoon Acceleration Speed (*PAS*), Platoon Speed Limit (*PSL*), and Platoon Real-time Speed (*PRS*). The platoon’s speed characteristics are represented as: *PS* = [*PAS, PSL, PRS*]. In this scenario, the *PS* of Platoon_A is [2 m/s^2^, 160 km/h, 90 km/h], and the *PS* of Platoon_B is [2 m/s^2^, 160 km/h, 100 km/h].
(11) The platoon destination is not the destination of each vehicle; it is set to guide the platoon and is generally updated in the weaving area or service area.

Algorithm 4 is a comprehensive algorithm that completely describes the whole process of vehicles and platoons on the basic road section by calling the specific algorithm in Section 3.3. This algorithm focuses on the change in the set and gives specific numerical changes.
**Algorithm 4:** A algorithm in the basic road section
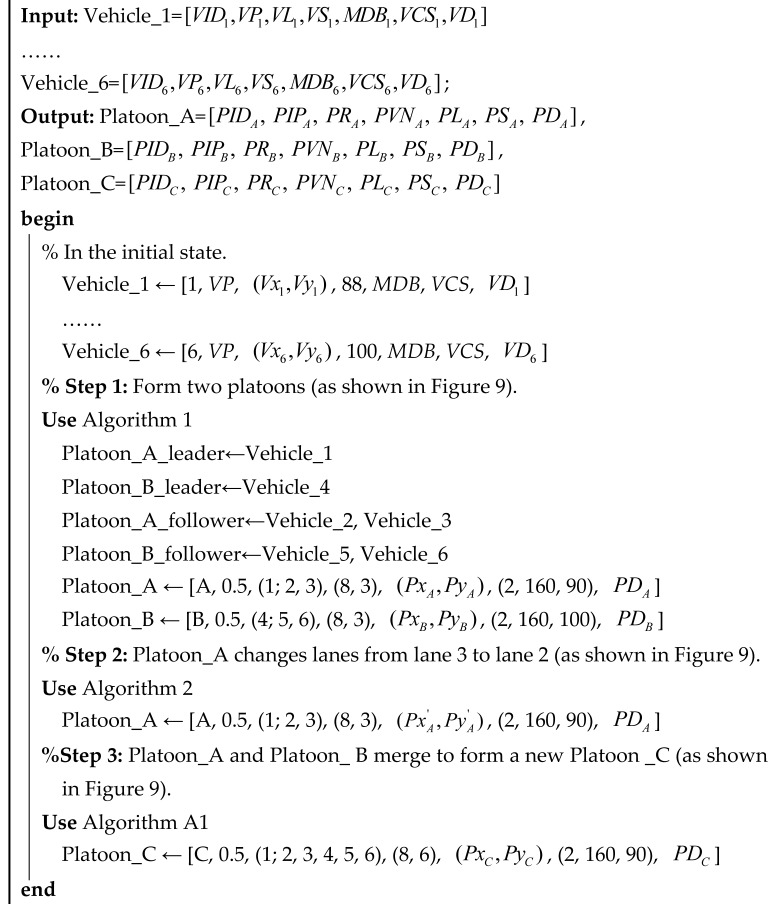


### 4.2. Scenario 2: In an Intersection

**Figure 10:**
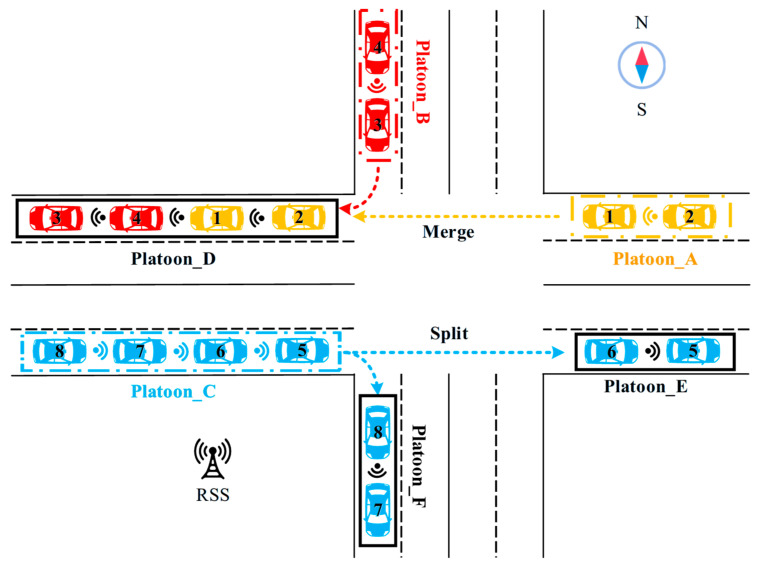
The complete life cycle in the intersection.

In the intersection scenario, three basic scenarios are considered to demonstrate the life cycle evolution process. First is the platoon formation process, where two isolated vehicles at the east approach, having the same destination and meeting the platoon formation conditions, form Platoon_A before reaching the intersection. During the merging process, assume that a two-vehicle platoon, Platoon_B, at the north approach is turning right at the intersection. Subsequently, Platoon_A, traveling straight through the intersection, merges with Platoon_B to form a new Platoon_D. During the splitting process, the four vehicles in Platoon_C split into two new platoons, Platoon_E for straight traffic and Platoon_F for right-turning vehicles. The specific process flow is illustrated in Figure 10.

The changes in the vehicle set and platoon set in the intersection of the life cycle are as follows:

**In the initialization phase (platooning):**

(1) At the east approach, there are two isolated vehicles with unique IDs (simplified as 1 and 2) and Vehicle Communication System (*VCS*). The Microscopic Driving Behavior (*MDB*) is obtained from the Road Side System (RSS), which provides lane-changing or car-following strategies. The speeds of the two vehicles are 48 km/h and 55 km/h, respectively (not exceeding the road speed limit). It is assumed that both vehicles intend to travel straight through the intersection, thus having the same Vehicle Destination (*VD*). In summary, Vehicle_1 = [1, *VP*, $\left(Vx_{1},Vy_{1}\right)$, 48, *MDB*, *VCS*, VD] and Vehicle_2 = [2, *VP*, $\left(Vx_{2},Vy_{2}\right)$, 55, *MDB*, *VCS*, VD].
(2) Two vehicles form Platoon_A, with a 2-m space headway, where Vehicle_1 serves as the platoon leader, so the Platoon Role (*PR*) of Platoon_A is (1; 2). The Platoon Maximum Vehicle Number (*PMN*) is assumed to be 8, and the Real-time Vehicle Number (*RVN*) is 2, so the Platoon Vehicle Number (*PVN*) of Platoon_A is (8, 2). The intersection speed limit is 50 km/h. Therefore, Platoon_A = [A, 2, (1; 2), (8, 2), $\left(Px_{A},Py_{A}\right)$, 50, PD].

**In the merging phase (when platoons merge):**

At the north approach of the intersection, a two-vehicle platoon, Platoon_B, is turning right at the intersection, represented as Platoon B = [B, 2, (3; 4), (8, 2), $\left(Px_{B},Py_{B}\right)$, 40, *PD*]. Then, Platoon_A, traveling straight through the intersection at the east approach, merges with Platoon_B to form a new Platoon_D. When Platoon_A and Platoon _B merge to form the new Platoon_D, the lead vehicle of Platoon_A (Vehicle_1) changes its role to a follower in the new platoon, while Vehicle_3 continues to serve as the leader of the newly merged four-vehicle Platoon_D. The speed of the new Platoon_D is 40. Therefore, Platoon_D = [D, 2, (3; 4, 1, 2), (8, 4), $\left(Px_{D},Py_{D}\right)$, 40, *PD*].

**In the split phase** **:**

Platoon_C splits into two new platoons, Platoon_E for straight traffic and Platoon_F for right-turning traffic. Platoon_C = [C, 2, (5; 6, 7, 8), (8, 4), $\left(Px_{C},Py_{C}\right)$, 45, $PD_{C}$]. The new Platoon_E = [E, 2, (5; 6), (8, 2), $\left(Px_{E},Py_{E}\right)$, 50, $PD_{E}$], and Platoon_F = [F, 2, (7; 8), (8, 2), $\left(Px_{F},Py_{F}\right)$, 30, $PD_{F}$].

Algorithm 5, similar to Algorithm 4, is also a comprehensive algorithm that accomplishes the whole evolution process of vehicles/platoons at intersections by invoking different sub-algorithms.
**Algorithm 5:** A comprehensive algorithm in the intersection
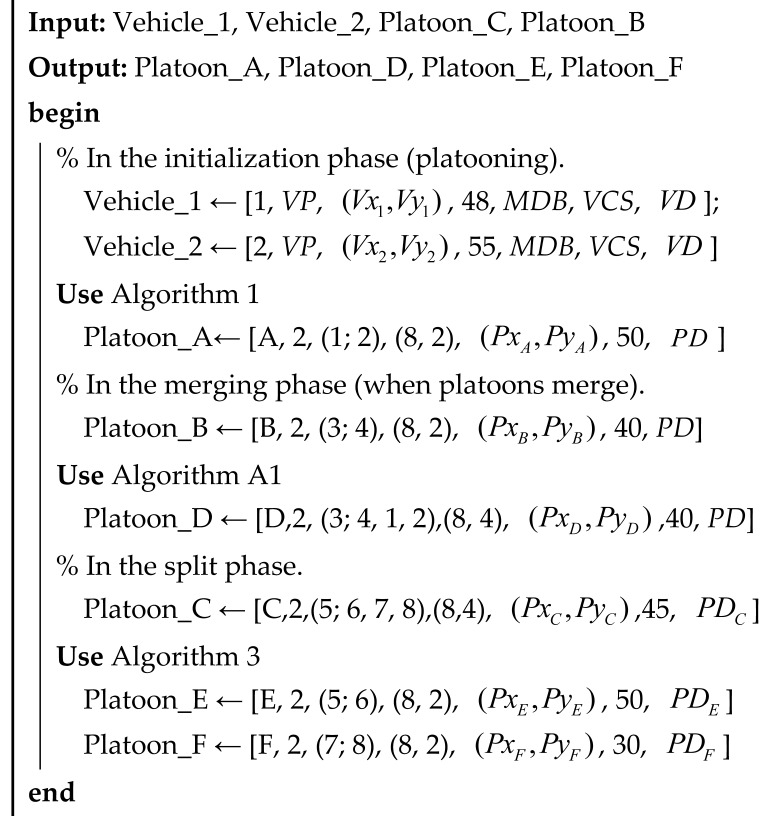


### 4.3. Experimental Validation

To validate the effectiveness of the proposed method, we conducted repeated platoon merging and splitting simulations using SUMO (v1.24.0) and Python (v3.7) on a workstation equipped with an Intel Core i7-13700H CPU @ 2.40 GHz, 32 GB RAM, and an NVIDIA RTX A500 GPU. We first configured an intersection traffic scenario and typical vehicle parameters; we then compared the method based on the proposed Minimum Platoon Set with SUMO’s default platoon management. Finally, we evaluated effectiveness using time consumption, energy consumption, CO_2_ emissions, and NOx emissions as performance metrics.

**Figure 11:**
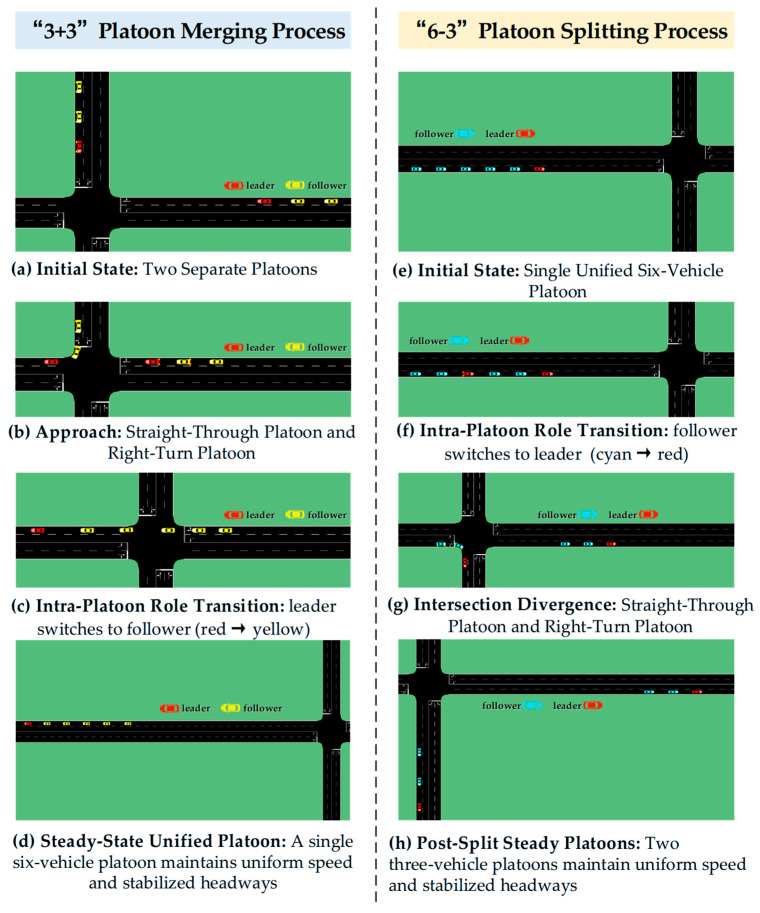
Schematic diagram of platoon merging and splitting processes.

**(1) Traffic Scenario:** The simulation environment comprises an unsignalized four-leg intersection with dual-lane approaches on each leg, as illustrated in Figure 11. This configuration enables two typical platoon coordination scenarios: (i) merging (“3+3”), in which two independent three-vehicle platoons approaching from orthogonal directions converge onto a common roadway and form a unified six-vehicle platoon via straight-through and right-turn maneuvers, respectively, and (ii) splitting (“6-3”), in which a unified six-vehicle platoon diverges at the intersection into two independent three-vehicle platoons following distinct routes. This layout represents a basic scenario for demonstrating dynamic role transitions and information exchange defined in the Minimum Platoon Set framework. These two representative cases are designed to demonstrate the platoon dynamic evolution process; subsequent quantitative evaluation will be conducted across a broader range of platoon sizes and diverse formation scenarios, extending beyond these specific examples.

**(2) Experimental Parameters:** The parameterization used in the simulations is consistent with the Minimum Platoon Set. Vehicle-level quantities including vehicle length, acceleration and deceleration limits, and maximum speed define the *VP* component of the *VPS* and bound individual-vehicle dynamics. Platoon-level quantities including the upper bound on space headway, the time headway, and a safety distance interpreted as the lower bound on space headway define the *PIP* within the *PPS* and serve as spacing targets during coordination. The maximum platoon size specifies the *PVN* capacity *PMN*, and the realized vehicle count in each run corresponds to *RVN*. These definitions are mutually consistent: headway targets govern operational gaps, the safety distance provides a lower constraint, and vehicle performance limits bound attainable speeds without contradicting the headway requirements. Numerical values are provided in Table 7.

**Figure 12:**
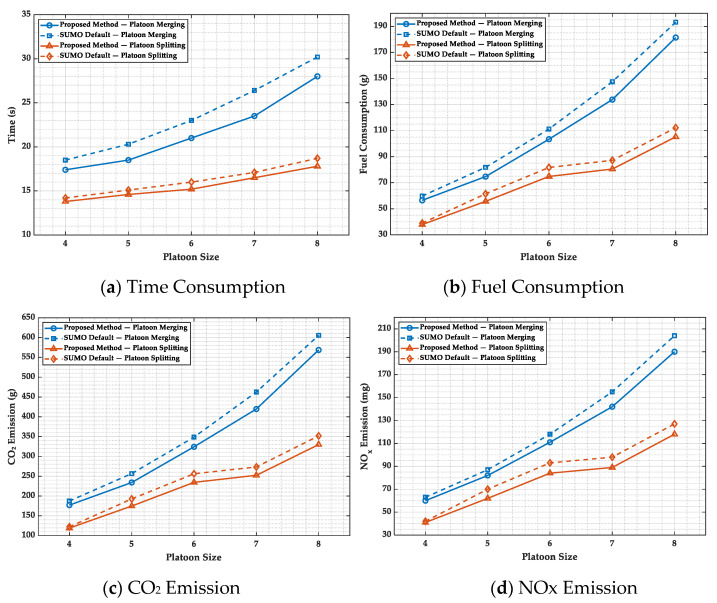
Performance comparison between *MPS* and SUMO default approaches.

**(3) Evaluation Scenarios and Metrics:** We evaluate platoon sizes from four to eight with merging patterns 2 + 2, 2 + 3, 3 + 3, 3 + 4, and 4 + 4 and splitting patterns 4 − 2, 5 − 2, 6 − 3, 7 − 3, and 8 − 4 (Table 8). For merging, the measurement period begins at the Intra-Platoon Role Transition where the catch-up platoon’s leader switches to follower (red→yellow) and ends when the unified platoon exhibits stable uniform speed and constant intra-platoon headways. For splitting, the measurement period begins at the Intra-Platoon Role Transition where a follower becomes the new leader of the diverging group (cyan→red) and ends when each resultant independent platoon exhibits stable uniform speed and constant intra-platoon headways with vehicles committed to their designated routes. The reported metrics are time consumption (period duration), fuel consumption, CO_2_ emissions, and NOx emissions, computed as cumulative sums over the vehicles within the corresponding period; Figure 12 presents results for both the proposed method and the SUMO default across the tested platoon sizes.

The experimental results demonstrate that all four metrics, including time consumption, fuel consumption, CO_2_ emissions, and NOx emissions, exhibit an upward trend as platoon size increases from 4 to 8 vehicles, with merging operations consistently requiring higher consumption than splitting operations. Compared to SUMO’s default method, the approach based on the Minimum Platoon Set achieves superior performance across all metrics, maintaining stable performance advantages across different platoon sizes. Specifically, in terms of time consumption, the proposed method reaches steady state more rapidly; regarding fuel consumption and emission metrics, our method maintains consistent performance advantages across all platoon sizes. Although time differences in splitting scenarios are relatively small, fuel consumption and emissions still show significant improvements. These enhancements primarily stem from the standardized information management framework provided by the Minimum Platoon Set, which reduces redundant information and enables more efficient and orderly coordination among vehicles, thereby minimizing ineffective operations caused by information processing burdens and ultimately reducing overall energy consumption and emission levels.

## 5. Conclusions and Future Directions

Vehicle platooning has the potential to significantly improve traffic operations. The development of platooning requires a comprehensive information set to describe the necessary elements for implementing vehicle platooning. By analyzing the attributes of vehicles and platoons, as well as the functions presented in state-of-the-art research on vehicle platooning, this paper proposes the Minimum Platoon Set to implement vehicle platooning. The Minimum Platoon Set includes measurables, communication factors, and operating instructions. The feasibility of the Minimum Platoon Set has been demonstrated by describing the attributes and functions of vehicles and platoons presented in several studies using the proposed set in Table 4 and Table 5. Furthermore, Section 4.1 and Section 4.2 present scenario demonstrations that show how the Minimum Platoon Set can describe the complete lifecycle of platoons in typical highway and intersection settings. In addition, Section 4.3 provides simulation-based validation in SUMO, where the proposed approach shows consistent advantages over the default method in time consumption, fuel consumption, and CO_2_/NOx emissions across multiple platoon sizes.

The Minimum Platoon Set can mitigate interoperability issues among automated vehicles from different manufacturers. The proposed Minimum Platoon Set is grounded in current studies on vehicle platooning and can be revised or extended to accommodate specialized applications as needed in the future.

The platoon management discussed in this paper focuses on platoon information management rather than control or communication management. The collected information forms the Minimum Platoon Set, which describes the platoon’s status and transformations through information presentation and temporal changes. The Minimum Platoon Set can serve as a foundational template for platoon management systems, enabling transportation authorities to derive insights and implement practical applications.

The Minimum Platoon Set proposed in this study constitutes an information management framework primarily intended to support the development and implementation of platoon management systems. In practical deployment, this framework needs to be integrated with specific communication protocols, security mechanisms, and control strategies to form a complete system. Future research will validate the performance of this information set across diverse traffic scenarios through large-scale simulation experiments and conduct quantitative comparisons with existing approaches to evaluate its effectiveness and efficiency under varying operational conditions.

Building on the advantages of the Minimum Platoon Set proposed herein for fleet information management, future research will introduce hierarchical clustering and distributed management architectures, in conjunction with edge computing and intelligent scheduling strategies, to mitigate communication bottlenecks and latency and enhance the scalability of the framework in dense Internet of Vehicles environments. Concurrently, robust security mechanisms, including encrypted communication protocols, mutual authentication schemes, and anomaly detection algorithms, will be developed to guard against identity spoofing, message tampering, and denial-of-service attacks. Moreover, the practical feasibility of the framework will be rigorously evaluated through large-scale simulations and field trials under real-world challenges such as heterogeneous V2X hardware interoperability and network synchronization delays. Finally, the effectiveness and robustness of the framework will be quantitatively validated across varying traffic densities and network load conditions, with the aim of laying a solid foundation for its deployment in intelligent transportation systems.

## Figures and Tables

**Table 1:** Different interpretations of the vehicle platoon instruction set.

Permission	*CV*	*VI*	*SM*	*IU*	*Maintain*	*Change*	*Adopt*
Follower	○	Verify	To leader	○	*	*	*
Leader	○	Assignment	To followers	○	○	○	○

○: As both the decision-maker and executor of the instructions; *: As the executor of the instructions.

**Table 2:** The declaration of vehicle information sharing.

Information Sharing	*VID*	*VP*	*VL*	*VS*	*MDB*	*VCS*	*VD*
To platoon leader	√	√	√	√	√	√	√
To another follower	√	×	√	×	×	√	×

√: The information item will be shared/transmitted; ×: The information item will **not** be shared/transmitted.

**Table 3:** The declaration of the information update cycle.

Update Cycle	*PID*	*PIP*	*PR*	*PVN*	*PL*	*PS*	*PD*
Real-time	F	F	F	F	T	T	F
State change	T	T	T	T	F	F	T

T: The information is updated according to the specified cycle; F: The information is not updated according to the specified cycle.

**Table 4:** Comparison with related works (compared with property set).

Ref.	Research Field (Type)	Main Elements/Function	Mapping of Property Set	Explanation
[14]	CACC (Cooperative Adaptive Cruise Control)	Lead vehicle/ Preceding vehicle	In the platoon set (Platoon Role)	Referring to the same thing.
[16]	Platoon control strategy	Speed/Acceleration/Distance/Time gap	In the platoon set (Platoon Internal Parameters/Platoon Speed)	It is extracted and summarized in this article.
[26]	Platoon control strategy	Platoon length/ Platoon speed/ Platoon acceleration/ Platoon gap	In the platoon set (Platoon Vehicle Number/Platoon Internal Parameters/Platoon Speed)	A property that can be substituted or derived.
[15]	Platoon control strategy	OBU (On-Board Unit)/CACC (Cooperative Adaptive Cruise Control)	In both the platoon and vehicle sets	Synthetic properties or formulas can be calculated and deduced by two sets.
[27]	Stochastic platooning strategy	Coordination strategy/ Optimal policy	In both the platoon and vehicle sets	Synthetic properties or formulas can be calculated and deduced by two sets.
[19,23]	Research on the specific scenario	Platoon attribute matrix/Trajectory	In vehicle set	“Time step” is added to the instruction set.
[18]	Platoon model/algorithm	Vehicle model/Platoon Configurations	In vehicle set (Vehicle Performance/Vehicle Location)	A parameter about model fit is not an essential element of the functional implementation of the vehicle platoon.

**Table 5:** Comparison with related works (compared with instruction set).

Ref.	Core Functionality	Classification	Involved Instruction	Achievement
[28]	Creation/Join/Maintain/Dissolution	Control set	All	Y
[29]	Route planning system	Communication set	All	Y
[30]	MPF Topology	Communication set	All	Y
[31]	D2D transmission	Communication set	*SM* (Sending the Message) and *IU* (Information Update)	Y
[32]	Platoon merge on-ramp	Control set	All	Y
[33]	Platoon mode in an intersection	Control set	All	Y
[12]	Platoon formation and dissolution in multi-lane highways	Control set	All	Y

Y: The core functionalities discussed in the respective studies can be realized by the corresponding instructions within the minimum instruction set proposed in this paper.

**Table 6:** Mapping between information elements and essential platoon functions.

Information Element	Essential Function(s) Requiring This Element	Consequence of Removal
*VID*	Vehicle identification in all operations	Cannot distinguish individual vehicles
*VP*	Leader selection, performance matching	Cannot assess compatibility or assign roles
*VL*	Spatial relationship determination, formation feasibility	Cannot determine relative positions or proximity
*VS*	Speed synchronization, platoon stability	Cannot coordinate velocities
*MDB*	Behavior prediction, safety assessment	Cannot anticipate vehicle actions
*VCS*	Communication capability verification	Cannot establish information exchange
*VD*	Route compatibility verification	Cannot ensure shared trajectory segments
*PID*	Platoon identity in multi-platoon scenarios	Cannot distinguish between platoons
*PIP*	Intra-platoon control parameters	Cannot maintain formation consistency
*PR*	Role assignment and authority management	Cannot organize hierarchical structure
*PVN*	Capacity constraint verification	Cannot prevent overloading
*PL*	Platoon positioning and navigation	Cannot locate platoon as an entity
*PS*	Platoon-level speed coordination	Cannot manage collective motion
*PD*	Destination-based platoon planning	Cannot plan route-dependent operations

**Table 7:** Simulation parameter configuration.

Parameter	Value	Parameter	Value
Vehicle length	5 m	Maximum acceleration	3.0 m/s^2^
Maximum space headway	15 m	Maximum deceleration	3.5 m/s^2^
Minimum time headway	0.5 s	Maximum platoon size	8 Vehicles
Minimum safe distance	2.5 m	Maximum speed	13 m/s

**Table 8:** Experimental scenario configuration for platoon merging and splitting.

Platoon Size	Platoon Merging	Platoon Splitting
4	2 + 2	4 − 2
5	2 + 3	5 − 2
6	3 + 3	6 − 3
7	3 + 4	7 − 3
8	4 + 4	8 − 4

## Data Availability

The data in this work is available upon request.

## Appendix A

**Algorithm A1:** Create a platoon for the “m + n” type

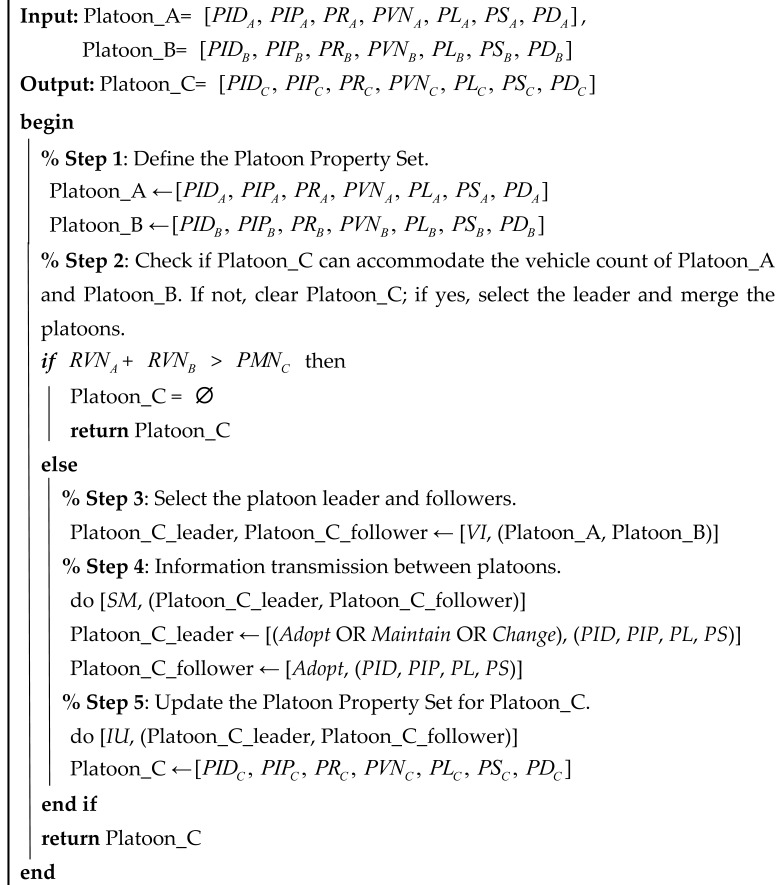

## Appendix B

**Algorithm A2:** Platoon dissolution
